# The additive effect of genetic and metabolic factors in the pathogenesis of nonalcoholic fatty liver disease

**DOI:** 10.1038/s41598-022-22729-5

**Published:** 2022-10-20

**Authors:** Yuya Seko, Kanji Yamaguchi, Kota Yano, Yusuke Takahashi, Kento Takeuchi, Seita Kataoka, Michihisa Moriguchi, Yoshito Itoh

**Affiliations:** Department of Molecular Gastroenterology and Hepatology, Graduate School of Medical Science, Kyoto Prefectural University of Medicine, Kawaramachi-Hirokoji, Kamigyou-Ku, Kyoto, 602-8566 Japan

**Keywords:** Gastroenterology, Medical research

## Abstract

Both genetic and metabolic factors influence the pathology of nonalcoholic fatty liver disease (NAFLD). The aim of this retrospective study was to evaluate the impact of these factors at each stage of disease. We analysed the impact of obesity, diabetes mellitus and genetic risk factors (alleles of *PNPLA3* or *HSD17B13*) on nonalcoholic steatohepatitis (NASH), significant fibrosis (stage ≥ 2) and advanced fibrosis (stage ≥ 3) in 346 patients. Genetic high risk was defined as having at least 2 risk alleles. The median age was 59 years, median body mass index was 27.1 kg/m^2^, and 46.8% had diabetes mellitus. Obesity was a risk factor for NASH, significant fibrosis, and advanced fibrosis. Diabetes mellitus increased the risk of NASH. Genetic risk increased the risk of significant and advanced fibrosis. Odds ratios for NASH, significant fibrosis and advanced fibrosis increased with the number of genetic and metabolic risk factors. The patients with both metabolic and genetic risks had an odds ratio of 12.30 for NASH, 5.50 for significant fibrosis, and 6.25 for advanced fibrosis. Factors strongly impact on the pathology of NAFLD differed according to the fibrosis stages. Synergistic effects were observed between genetic and metabolic factors at all stages.

## Introduction

Nonalcoholic fatty liver disease (NAFLD) is the leading cause of chronic liver disease worldwide^1^. NAFLD is recognised as the hepatic manifestation of metabolic syndrome, and it is commonly complicated by obesity, dyslipidaemia, hypertension and diabetes mellitus (DM)^2–4^. NAFLD encompasses a wide spectrum of liver pathology, ranging from non-alcoholic fatty liver (NAFL), which is usually benign, to non-alcoholic steatohepatitis (NASH), which is characterised by steatosis, lobular inflammation, and hepatocellular injury, and may progress to liver cirrhosis (LC), hepatic failure and hepatocellular carcinoma (HCC). Although fibrosis progression is slow in NAFL, each fibrosis stage in NASH progresses over 7 years^5^. Because hepatic fibrosis is the strongest predictor of liver-related events and mortality^6,7^, the prevention of fibrosis progression is an important issue. Cardiovascular disease is the leading cause of mortality, followed by cancer and then liver disease. Results of a large cohort study demonstrated that liver fibrosis increased the risk for cardiovascular disease among patients with NAFLD without LC^8^.

It is well known that obesity and metabolic syndrome, including DM, are associated with NASH and disease severity. The predictors of fibrosis progression are not clear, but higher liver enzyme levels, presence of DM, and family history of LC have been associated^9–11^. In a large-scale trial of 1365 Japanese patients with biopsy-proven NAFLD, fibrosis stage increased with age, and body mass index (BMI) and DM were positively correlated with fibrosis stage. In that study, multivariate analysis identified age and DM as significant risk factors for advanced fibrosis^12^. Genetic factors also affect NAFLD, at an estimated rate ranging from 35 to 61%^13,14^. Single-nucleotide polymorphisms (SNP) in patatin-like phospholipase domain-containing 3 (PNPLA3) are an independent risk factor for fibrosis through effects on hepatic stellate cells. In hepatic stellate cells, wild-type PNPLA3 hydrolyses retinyl esters to promote extracellular retinol release, but its activity is reduced in the presence of the I148M mutant, which leads to pro-fibrotic activity of the stellate cells^15,16^. A variant of 17-beta hydroxysteroid dehydrogenase 13 (HSD17B13) has a protective effect on this liver injury evoked by PNPLA3 I148M risk variant^17^. Though the mechanism of fibrosis is not clear, HSD17B13 is associated with increased steatosis and decreased hepatic inflammation^18^. We previously reported that carriage of the HSD17B13 rs6834314 G allele attenuated the effect of the PNPLA3 rs738409 GG genotype on advanced hepatic fibrosis in Japanese patients with NAFLD^19^. As mentioned above, both metabolic and genetic factors increase the risk for NAFLD progression. However, the differences in the magnitude of influence of each factor at each stage of NAFLD progression are not clear.

The aim of this retrospective study was to evaluate the impact of metabolic and genetic factors at each stage of NAFLD and to evaluate the synergistic effects of these factors.

## Material and methods

### Patients

We enrolled 346 Japanese biopsy-proven NAFLD patients at the Department of Gastroenterology and Hepatology, Kyoto Prefectural University of Medicine (Kyoto, Japan) from January 2013 to February 2020 in this study. We excluded the patients diagnosed viral hepatitis, autoimmune hepatitis, drug-induced liver disease and other liver disease. This study was approved by the Ethical Review Board of Kyoto Prefectural University of Medicine (ERB-C-1416). All patients provided written informed consent at the time of liver biopsy, and the study was conducted in accordance with the Declaration of Helsinki (2013).

### Physical examination, laboratory and clinical parameters

We collected laboratory data including platelet counts, albumin, total cholesterol, triglycerides, low density lipoprotein cholesterol, high-density lipoprotein cholesterol, fasting plasma glucose, haemoglobin A1c, AST, ALT, gamma-glutamyl transpeptidase and the 7S domain of type IV collagen. These parameters were measured using standard clinical chemical laboratory techniques.

Hypertension was diagnosed if the patient was receiving antihypertensive medication and/or had a resting recumbent blood pressure of at least 130/85 mmHg. Patients taking oral hypoglycaemic agents and those with a fasting glucose concentration > 126 mg/dL or a random glucose concentration > 200 mg/dL were diagnosed with DM. When the serum level of low-density lipoprotein cholesterol was greater than 140 mg/dL and/or the triglyceride level was more than 150 mg/dL, and/or high-density lipoprotein cholesterol level was less than 40 mg/dL, patients were diagnosed with dyslipidaemia. These diagnostic criteria were in accordance with previous studies^19^.

### DNA preparation and SNP genotyping

Genomic DNA was extracted from blood samples using the DNeasy Blood & Tissue kit (Qiagen). The SNPs rs738409 and rs6834314 were genotyped in each sample using TaqMan SNP genotyping assays (Applied Biosystems) with commercially available predesigned SNP-specific primers for PCR amplification and extension reactions, according to the manufacturer’s protocol. These measurement methods were in accordance with previous studies^19^. We divided patients according to their number of risk alleles (G allele of *PNPLA3* or A allele of *HSD17B13*). Patients who had at least two risk alleles were defined as a genetic high risk group.

### Liver histology

The liver biopsy specimens were stained with haematoxylin and eosin and Masson trichrome stain. The specimens were evaluated by hepatic pathologists who were blinded to the clinical findings. Grading and staging were performed as described by Kleiner et al.^20^ and Brunt et al.^21^. In this study, significant fibrosis was defined as fibrosis stages 2, 3 and 4, and advanced fibrosis was defined as fibrosis stages 3 and 4.

### Statistical analysis

Patient characteristics were assessed using the Chi-square test or the Mann–Whitney *U* test, depending on the distribution of the data. We performed logistic regression analysis to identify factors associated with significant and advanced fibrosis by calculating adjusted odds ratios (aORs) and 95% confidence intervals (CIs) after adjusting for age, obesity, DM, Hypertension, Hyper lipidemia and genetic risk. To assess the additive interaction, the Synergism index (S) proposed by Rothman was calculated^22^. All statistical analyses were performed using SPSS version 25 (SPSS Inc., Chicago, IL). All *p*-values less than 0.05 obtained from two-tailed tests were considered significant.

### Consent to participate

This study was approved by the institutional review board of Kyoto Prefectural University of Medicine, Kyoto, Japan (approval no. ERB-C-1416) and fully complied with the 1964 Helsinki declaration and its later amendments.

## Results

### Patients characteristics

Table 1 summarizes the demographic profile and laboratory and histological data of the study patients. Among the 346 subjects, the median age was 59 years, the median BMI was 27.1 kg/m^2^, and 176 (50.9%) were female; 244 cases (70.5%) were diagnosed with NASH. The number of cases with significant and advanced fibrosis was 121 (35.0%) and 62 (17.9%), respectively. The *PNPLA3* genotype frequencies were CC in 66 patients (19.1%); CG in 145 (41.9%); and GG in 135 (39.0%). The *HSD17B13* genotype frequencies were AA in 180 patients (52.0%); AG in 129 patients (37.3%); and GG in 37 patients (10.7%). Patients who had at least two risk alleles were defined as a genetic high risk group. The number of patients with a genetic risk of 0/1/2/3/4 was 7/42/105/116/76, respectively. Table 2 summarizes the characteristics of subjects according to genetic risk. The genetic high risk group contained more females and more older patients than the genetic low risk group. The serum levels of AST and type IV collagen 7 s were significantly higher in the genetic high risk group; in contrast, the platelet count and serum triglyceride levels were significantly higher in the genetic low risk group than in the genetic high risk group. There were no significant differences in BMI, prevalence of hypertension, DM, and hyperlipidaemia between the two groups.

**Table 1:** Patient characteristics.

	Patients (n = 346)
Female, n (%)	176 (50.9%)
NASH, n (%)	244 (70.5%)
Age, years	59 (22–84)
BMI, kg/m^2^	27.1 (17.6–45.2)
Hypertension, n (%)	147 (42.5%)
Diabetes mellitus, n (%)	162 (46.8%)
Hyperlipidemia, n (%)	198 (57.2%)
*PNPLA3*, CC/CG/GG, n	66/145/135
*HSD17B13*, AA/AG/GG, n	180/129/37
Albumin, g/dL	4.4 (3.5–5.3)
AST, IU/L	42 (12–208)
ALT, IU/L	55 (9–263)
GGT, IU/L	59 (13–716)
Platelet count, × 10^3^/μL	209.5 (57–450)
Total cholesterol, mg/dL	198 (95–347)
Triglycerides, mg/dL	139 (18–923)
LDL cholesterol, mg/dL	121 (36–259)
HDL cholesterol, mg/dL	51 (21–103)
FPG, mg/dL	109 (76–374)
HbA1c, %	6.2 (4.8–11.5)
Type IV collagen 7 s, ng/mL	4.9 (2.5–12.0)
Fibrosis stage, 0/1/2/3/4, n	115/110/59/43/19
Steatosis grade, 1/2/3, n	102/186/58
Inflammation grade, 0/1/2/3, n	14/202/110/20
Ballooning grade, 0/1/2, n	101/149/96

*BMI* body mass index, *AST* aspartate aminotransferase, *ALT* alanine aminotransferase, *GGT* gamma-glutamyl transferase, *LDL* low-density lipoprotein, *HDL* high-density lipoprotein, *FPG* fasting plasma glucose.

**Table 2:** Patient characteristics by genetic risk.

	Genetic low risk n = 154	Genetic high risk n = 192	*p*-value
Female, n (%)	88 (57.1%)	82 (42.7%)	0.009
NASH, n (%)	107 (69.5%)	137 (71.4%)	0.723
Age, years	56 (22–81)	60 (22–84)	0.013
BMI, kg/m^2^	27.3 (19.3–44.2)	26.8 (17.6–45.2)	0.166
Hypertension, n (%)	62 (40.3%)	85 (44.3%)	0.512
Diabetes mellitus, n (%)	71 (46.1%)	91 (47.4%)	0.829
Hyperlipidemia, n (%)	90 (58.4%)	105 (54.7%)	0.585
Albumin, g/dL	4.5 (3.6–5.3)	4.4 (3.5–5.2)	0.082
AST, IU/L	38 (12–192)	49 (13–208)	0.001
ALT, IU/L	51 (9–198)	57 (10–263)	0.084
GGT, IU/L	58 (14–416)	59.5 (13–716)	0.920
Platelet count, × 10^3^/μL	222 (57–420)	199.5 (65–450)	0.004
Total cholesterol, mg/dL	199 (123–347)	197 (95–301)	0.304
Triglycerides, mg/dL	145 (37–923)	127 (18–642)	0.012
LDL cholesterol, mg/dL	122.5 (36–259)	121 (36–197)	0.812
HDL cholesterol, mg/dL	51 (21–103)	51.5 (26–103)	0.842
FPG, mg/dL	109 (77–374)	109 (76–252)	0.877
HbA1c, %	6.2 (4.9–11.2)	6.1 (4.8–11.5)	0.689
Type IV collagen 7 s, ng/mL	4.5 (2.7–12.0)	5.2 (2.5–12.0)	< 0.01
Fibrosis stage, 0/1/2/3/4, n	62/51/26/11/4	53/59/33/32/15	0.005
Steatosis grade, 1/2/3, n	3/85/26	59/101/32	0.943
Inflammation grade, 0/1/2/3, n	8/102/39/5	6/100/71/15	0.014
Ballooning grade, 0/1/2, n	46/74/34	55/75/62	0.088

*BMI* body mass index, *AST* aspartate aminotransferase, *ALT* alanine aminotransferase, *GGT* gamma-glutamyl transferase, *LDL* low-density lipoprotein, *HDL* high-density lipoprotein, *FPG* fasting plasma glucose.

Although there was no significant difference between the two groups in the number of patients with NASH (*p* = 0.723), the numbers of patients with significant and advanced fibrosis were significantly greater in the genetic high risk group (41.7% and 24.5%, respectively) than those in the genetic low risk group (26.6 and 9.7%, respectively).

### Factors associated with histological findings

**Figure 1:**
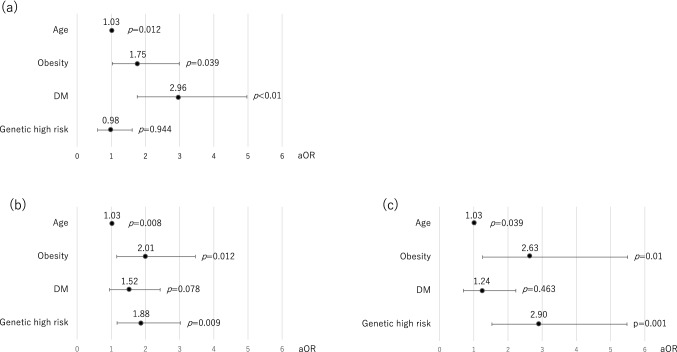
Adjusted odds ratios of factors for (**a**) NASH, (**b**) significant fibrosis and (**c**) advanced fibrosis.

To estimate the impact on histological findings, we examined the correlations of age, obesity, DM and genetic risk with NASH, significant fibrosis, and advanced fibrosis. Multivariate logistic regression analysis revealed that age was a risk factor for NASH (aOR 1.03, *p* = 0.012), significant fibrosis (aOR 1.03, *p* = 0.008), and advanced fibrosis (aOR 1.03, *p* = 0.039). Obesity increased the risk of NASH (aOR 1.75, p = 0.039), significant fibrosis (aOR 2.01, *p* = 0.012), and advanced fibrosis (aOR 2.63 *p* = 0.01). The presence of DM only increased the risk of NASH with aOR to 2.96 but did not increase the risk for both significant and advanced fibrosis. On the other hand, genetic high risk increased the risk for both significant fibrosis (aOR 1.88, *p* = 0.009) and advanced fibrosis (aOR 2.90, *p* = 0.001) (Table 3, Fig. 1). In describing steatosis grade, age was the only significant negative risk factor for both steatosis grade ≥ 2 and ≥ 3 (Supplementary Table 1).

**Table 3:** Multivariate logistic regression analysis of age, obesity, DM and genetic high risk.

	Non-NASH vs. NASH		stages 0–1 vs. stages 2–4		stages 0–2 vs. stages 3–4	
	aOR 95% CI	*p*	aOR 95% CI	*p*	aOR 95% CI	*p*
Age	1.03 (1.01–1.05)	0.012	1.03 (1.01–1.05)	0.008	1.03 (1.00–1.05)	0.039
Obesity	1.75 (1.03–2.99)	0.039	2.01 (1.16–3.46)	0.012	2.63 (1.26–5.50)	0.01
DM	2.96 (1.76–4.97)	< 0.01	1.52 (0.95–2.43)	0.078	1.24 (0.70–2.23)	0.463
Genetic high risk	0.98 (0.60–1.61)	0.944	1.88 (1.17–3.02)	0.009	2.90 (1.53–5.48)	0.001

*aOR* adjusted odds ratio, *CI* confidence interval, *DM* diabetes mellitus, *NASH* non-alcoholic steatohepatitis.

### The combined effect of genetic and metabolic factors

**Figure 2:**
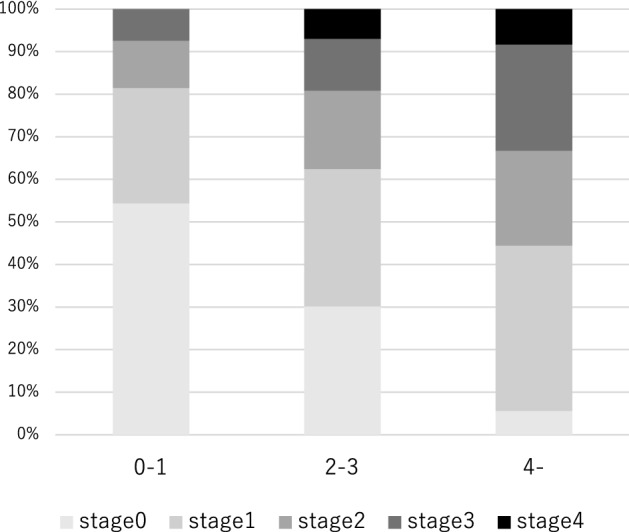
The prevalence of patients with each fibrosis stage according to the number of factors.

**Figure 3:**
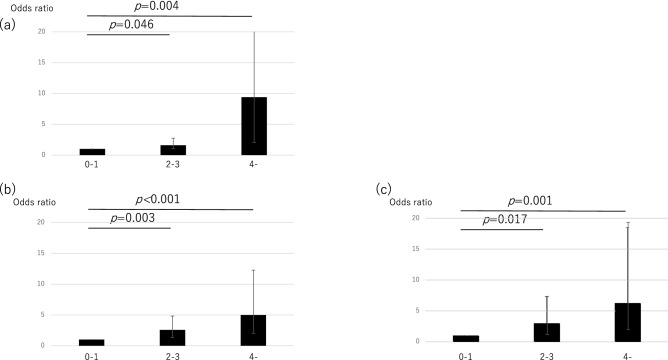
Odds ratios of each group for (**a**) NASH, (**b**) significant fibrosis and (**c**) advanced fibrosis.

Next, we assessed the combined effect of genetic and metabolic factors on advanced fibrosis. Table 4 shows the joint effects of the presence of DM, obesity, and genetic risk. The estimated synergism index (S) was greater than 1, indicating a departure from additivity in the joint effect of genetic risk and DM (S = 1.12) and in the joint effect of genetic risk and obesity (S = 1.57). This indicated that genetic risk, may exacerbate the effect of DM and obesity on advanced fibrosis (Table 4). Factors of age > 60 years, obesity, DM, and genetic high risk were chosen as risk factors and divided into three levels according to the number of factors possessed. Patients with up to one risk factor were defined as low group, those with two to three risk factors as middle group, and those with four risk factors as high group. The prevalence of patients with advanced fibrosis was 33.3% for high group and significantly greater than that for low group (7.4%) and middle group (19.2%) (Fig. 2). Compared with low group, middle group had an OR of 1.60 (95% CI 1.06–2.74, *p* = 0.046) for NASH, an OR of 2.57 (95% CI 1.37–4.83, *p* = 0.003) for significant fibrosis, and an OR of 2.99 (95% CI 1.21–7.37, *p* = 0.017) for advanced fibrosis. Compared with low group, high group had an OR of 9.40 (95% CI 2.06–42.84, *p* = 0.004) for NASH, an OR of 4.99 (95% CI 2.03–12.28, *p* < 0.001) for significant fibrosis, and an OR of 6.25 (95% CI 2.02–19.35, *p* = 0.001) for advanced fibrosis (Table 5, Fig. 3). The number of these risk factors was unrelated to steatosis grade (Supplementary Table 2).

**Table 4:** Interaction of genetic factor with diabetes mellitus and obesity on advanced fibrosis.

Interaction Variables		p value	OR (95% CI)	S
DM	Genetic risk			
Negative	Negative		1	
Positive	Negative	0.261	1.86 (0.63–5.52)	
Negative	Positive	0.009	3.57 (1.37–9.29)	
Positive	Positive	0.001	4.86 (1.88–12.56)	1.12
Obesity	Genetic risk			
Negative	Negative		1	
Positive	Negative	0.095	3.86 (0.79–18.85)	
Negative	Positive	0.186	2.81 (0.61–13.03)	
Positive	Positive	0.005	8.31 (1.92–36.06)	1.57

*CI* confidence interval, *OR* odds ratio, *DM* diabetes mellitus, *S* Synergy Index.

**Table 5:** Multivariate logistic regression analysis by the number of factors.

Number of factors	Non-NASH vs. NASH		stages 0–1 vs. stages 2–4		stages 0–2 vs. stages 3–4	
	OR 95% CI	*p*	OR 95% CI	*p*	OR 95% CI	*p*
0–1	1		1		1	
2–3	1.60 (1.06–2.74)	0.046	2.57 (1.37–4.83)	0.003	2.99 (1.21–7.37)	0.017
4-	9.40 (2.06–42.84)	0.004	4.99 (2.03–12.28)	< 0.001	6.25 (2.02–19.35)	0.001

*CI* confidence interval, *NASH* non-alcoholic steatohepatitis, *OR* odds ratio.

## Discussion

In the present study, we investigated the effect of metabolic factors, such as obesity and DM, and genetic factors on the pathology of NAFLD. Kanwal et al. showed that metabolic abnormalities (obesity, hypertension, diabetes, and lipid abnormalities) were significantly associated with the development of cirrhosis and HCC in a large US cohort of patients with NAFLD, and the level of risk increased with the number of metabolic abnormalities^23^. Several studies have found that mutation in the genes associated with hepatic lipid metabolism, including *PNPLA3*, predispose to the development of NAFLD, hepatic fibrosis, and HCC in the presence of a metabolic disorder condition^24,25^. Conversely, a splice variant in *HSD17B13* prevents fibrosis and HCC development^17^ A combination analysis of genes was also performed. Doniovanni et al. developed a polygenic risk score based on the relationship of *PNPLA3*, *TM6SF2*, *GCKR* and *MBOAT7* with hepatic fat content and showed that the impact of genetic risk variants on fibrosis is proportional to that on hepatic fat^26^. Another study reported that the combination of *PNPLA3*, *TM6SF2* and *HSD17B13* predicted NASH-cirrhosis and HCC in general population settings^27^. In this study, we determined genetic risk using *PNPLA3* and *HSD17B13*, because *PNPLA3* genotypes affect histological features, including fibrosis, but the *TM6SF2* genotype does not affect histological features in Japanese patients with NAFLD^28^.

We found that metabolic and genetic factors have different effects on each step in the pathogenesis of NAFLD. To our knowledge, the present study is the first to demonstrate that metabolic disorder mainly affects the early stage of NAFLD and genetic factors affect significant to advanced fibrosis. Age and obesity contributed at all steps, from NASH development to advanced fibrosis. These results indicate that obesity is the basis for the pathological progression of NAFLD and that its effects accumulate with age. DM had a strong effect on the development of NASH (aOR 2.96) but no significant effect on fibrosis progression. On the other hand, genetic factors did not show a significant impact on NASH development but increased the risk for significant and advanced fibrosis (aOR 1.88 and 2.90, respectively). Interpreting this result is difficult. The genetic factors, even *PNPLA3*, by themselves would not be expected to have enough impact to worsen the NAFLD condition. PNPLA3 was reported to operate as a triacylglycerol hydrolase and as an acylglycerol transacylase^29^, but the mechanism by which this polymorphism, which attenuates hydrolase activity^30^, increases liver fat is unclear. One plausible mechanism is that the I148M variant is resistant to ubiquitylation and proteasomal degradation, disrupting triglyceride mobilization to promote its accumulation and PNPLA3 I148M interferes with adipose triglyceride lipase (ATGL) activity by interacting with its cofactor^31,32^. Thus, PNPLA3 I148M may cause the progression of NAFLD with no or few metabolic abnormalities. Furthermore, PNPLA3 is also known to cause an independent increase in fibrosis risk through its effect on hepatic stellate cells. In hepatic stellate cells, PNPLA3 normally hydrolyses retinyl esters to promote the release of retinol to the extracellular space, but the presence of the I148M mutant reduces its activity, leading to increased fibrosis in stellate cells^15,16^. It is not clear why diabetes was not involved in fibrosis progression. A previous study reported that the prevalence of DM was associated with fibrosis severity^12^. The differences between that study and our study may have based on the difference in the prevalence of advanced fibrosis.

In addition, we evaluated the combined effect of genetic and metabolic factors on liver histology. We divided patients into three grades according to the number of metabolic and genetic risk factors they had. The prevalence of patients with advanced fibrosis gradually increased from 7.4% in low group to 33.3% in high group. The risk of NASH, significant fibrosis, and advanced fibrosis was significantly greater in high group than in low group. This finding aligns with previous evidence that the effect of PNPLA3 on liver injury is stronger in patients with higher metabolic risk^26^ and that patients with DM carrying the PNPLA3 G-allele had higher circulating free fatty acid concentrations and greater adipose tissue insulin resistance than noncarriers^33^. A recent study by Israelsen et al. showed that genetic factors (*TM6SF2* and *PNPLA3*) increased the risk of clinical and metabolic traits for fibrosis in patients with alcohol-related liver disease^34^. Pennisi et al. developed a genetic and metabolic staging scoring system and showed its effectiveness for prediction of liver-related events^35^. They created a formula with sex, age, DM, high-density lipoprotein cholesterol, albumin, platelet count, PNPLA3, TM6SF2, and HSD17B13 from the weighted sum of the risk factors. The risk of liver-related events increased from 4% in the best group to 91% in the worst group. Our results also demonstrated the usefulness of combining genetic and metabolic factors.

There are several limitations to the present study. The small number of patients without NASH may overestimate the predictive power. Also, the number of patients in g high group was relatively lower than in low and middle group. We have not been able to examine the impact of insulin resistance, epigenetics and nutrigenetics in this study. Furthermore, the gut microbiome is also an important factor associated with NASH pathology. The score was created without considering the specific weight of the genetic and metabolic factors. Since this study was conducted on Japanese patients only, the impact of genetic factor may be overestimated. It is necessary to examine whether similar results can be obtained in other racial groups with different *PNPLA3* and *HSD17B13* prevalence. On the other hand, by limiting the genetic risk to two genes, the present predictive model is simpler than previous models. We believe that the diagnostic method of NAFLD and the inclusion of a relatively large number of patients with fibrosis development also enhanced the accuracy of the study.

In conclusion, factors that most influenced the pathology of NAFLD differed depending on the stage of disease. Metabolic factors have a strong influence early in the course of NAFLD, and genetic factors promoted fibrosis progression. Even in the early stages, synergistic effects were observed between genetic and metabolic factors. Control of DM and obesity is important for preventing disease progression.

## Supplementary Information

Supplementary Tables.

## Data Availability

The datasets generated and/or analysed during the current study are not publicly available due to ethical restrictions imposed by an IRB but are available from the corresponding author on reasonable request.
