# Supplementary material for: The additive effect of genetic and metabolic factors in the pathogenesis of nonalcoholic fatty liver disease
Source: Sci Rep. 2022 Oct 20;12:17608. doi: 10.1038/s41598-022-22729-5 (PMC9584936; doi:10.1038/s41598-022-22729-5)
Supplement: Supplementary file 1 — Supplementary Tables. [file 41598_2022_22729_MOESM1_ESM.docx]

Supplement Table 1. Multivariate logistic regression analysis of age, obesity, DM and genetic high risk

|  | steatosis 0-1 vs steatosis 2-3 | | steatosis 0-2 vs steatosis 3 | |
| --- | --- | --- | --- | --- |
|  | aOR 95% CI | *p* | aOR 95% CI | *p* |
| Age | 0.45 (0.27-0.73) | 0.001 | 0.17 (0.08-0.35) | <0.01 |
| Obesity | 1.40 (0.84-2.34) | 0.200 | 0.53 (0.27-1.02) | 0.057 |
| DM | 1.06 (0.65-1.71) | 0.829 | 1.41 (0.77-2.57) | 0.262 |
| Genetic high risk | 0.80 (0.58-1.52) | 0.796 | 1.13 (0.62-2.04) | 0.692 |

aOR, adjusted odds ratio; CI, confidence interval; DM, diabetes mellitus; NASH, non-alcoholic steatohepatitis.

Supplement Table 2. Multivariate logistic regression analysis by the number of factors.

|  | steatosis 0-1 vs steatosis 2-3 | | steatosis 0-2 vs steatosis 3 | |
| --- | --- | --- | --- | --- |
| Number of factors | OR 95% CI | *p* | OR 95% CI | *p* |
| 0-1 | 1 |  | 1 |  |
| 2-3 | 0.90 (0.51-1.59) | 0.72 | 0.75 (0.40-1.42) | 0.375 |
| 4- | 0.66 (0.29-1.53) | 0.331 | 0.34 (0.094-1.25) | 0.105 |

CI, confidence interval; NASH, non-alcoholic steatohepatitis; OR, odds ratio.
